# Effectiveness of the Ready to Reduce Risk (3R) complex intervention for the primary prevention of cardiovascular disease: a pragmatic randomised controlled trial

**DOI:** 10.1186/s12916-020-01664-0

**Published:** 2020-07-27

**Authors:** Jo L. Byrne, Helen M. Dallosso, Stephen Rogers, Laura J. Gray, Ghazala Waheed, Prashanth Patel, Pankaj Gupta, Yvonne Doherty, Melanie J. Davies, Kamlesh Khunti

**Affiliations:** 1 Leicester Diabetes Centre, University Hospitals of Leicester NHS Trust, Leicester, UK; 2 Department of Health Sciences, University of Leicester, Leicester, UK; 3 Innovation and Research Unit, Northamptonshire Healthcare NHS Foundation Trust, Northampton, UK; 4 Diabetes Research Centre, Leicester General Hospital, University of Leicester, Leicester, UK; 5 Department of Cardiovascular Sciences, University of Leicester, Leicester, UK; 6 Department of Clinical Pathology and Metabolic Sciences, University Hospitals of Leicester NHS Trust, Leicester, UK; 7 York Diabetes Centre, York Teaching Hospital NHS Foundation Trust, York, UK

**Keywords:** Cardiovascular disease, Medication adherence, Patient education, Complex interventions, Statins, Lifestyle interventions, Behaviour interventions

## Abstract

**Background:**

Cardiovascular disease is responsible for 31% of all global deaths. Primary prevention strategies are needed to improve longer-term adherence to statins and healthy lifestyle behaviours to reduce risk in people at risk of cardiovascular disease.

**Methods:**

Pragmatic randomised controlled trial recruited between May 2016 and March 2017 from primary care practices, England. Participants (*n* = 212) prescribed statins for primary prevention of cardiovascular disease with total cholesterol level ≥ 5 mmol/l were randomised: 105 to the intervention group and 107 to the control group, stratified by age and sex. The 3R intervention involved two facilitated, structured group education sessions focusing on medication adherence to statins, lifestyle behaviours and cardiovascular risk, with 44 weeks of medication reminders and motivational text messages and two supportive, coaching phone calls (at approximately 2 weeks and 6 months). The control group continued with usual clinical care. Both groups received a basic information leaflet. The primary outcome was medication adherence to statins objectively measured by a biochemical urine test. Self-reported adherence and practice prescription data provided additional measures. Secondary outcomes included cholesterol profile, blood pressure, anthropometric data, cardiovascular risk score, and self-reported lifestyle behaviours and psychological measures (health/medication beliefs, quality of life, health status). All outcomes were assessed at 12 months.

**Results:**

Baseline adherence to statins was 47% (control) and 62% (intervention). No significant difference between the groups found for medication adherence to statins using either the urine test (OR 1.02, 95% CI 0.34 to 3.06, *P* = 0.968) or other measures. This may have been due to the higher than expected adherence levels at baseline. The adjusted mean difference between the groups (in favour of the intervention group) for diastolic blood pressure (− 4.28 mmHg (95% CI − 0.98 to − 1.58, *P* = 0.002)) and waist circumference (− 2.55 cm (95% CI − 4.55 to − 0.55, *P* = 0.012)). The intervention group also showed greater perceived control of treatment and more coherent understanding of the condition.

**Conclusions:**

The 3R programme successfully led to longer-term improvements in important clinical lifestyle indicators but no improvement in medication adherence, raising questions about the suitability of such a broad, multiple risk factor approach for improving medication adherence for primary prevention of CVD.

**Trial registration:**

International Standard Randomized Controlled Trial Number (ISRCTN16863160), March 11, 2006.

## Background

In 2016, approximately 17.9 million people died of cardiovascular disease (CVD) which represented 31% of all global deaths [1]. It is estimated that 80% of such deaths are caused by preventable, premature heart disease and stroke [2]. International guidelines for the primary prevention of CVD provide evidence-based recommendations with regard to lifestyle factors (diet, exercise, weight, smoking and alcohol) and statins to reduce risk and prevent these diseases [3–5]. The general consensus is that, despite some differences in the detail of the recommendations, there are benefits of exercise, cessation of smoking and statins for people at high risk of CVD. Regarding statin treatment, high-intensity statins are specifically recommended for the primary prevention of CVD in the United Kingdom (UK) [3] and the United States of America (USA) [4]. In the UK, the recommendation is to offer a high-intensity statin for the primary prevention of CVD to people who have a 10% or greater 10-year risk of developing CVD, assessed by using the QRISK2 assessment tool [6]; in the most recent guidance published in the USA, a high-intensity statin is recommended, without any CVD risk assessment, for people aged between 20 and 73 years with a low-density lipoprotein cholesterol (LDL-C) level ≥ 190 mg/dl (4.9 mmol/l).

Against this background, health promotion interventions have focused on modifying lifestyle behaviours as a logical first step to reduce CVD risk, and multiple risk factor interventions (ones that focus on several risks at the same time by offering structured, comprehensive lifestyle education) are generally considered to be the best approach [7]. Such interventions have been shown to have an effect on some risk factors, especially systolic blood pressure, diastolic blood pressure, body mass index and waist circumference [7]. However, there have been very few multiple risk factor interventions for CVD primary prevention that have medication adherence to statins as a primary aim [8, 9].

Estimated rates of adherence to cardiovascular medication range from 50 to 60% [10, 11]. In particular, rates of non-adherence to statins, when compared to other CVD preventative medications, are much higher [12], and poor adherence to statins (amongst primary prevention patients) is associated with an increased risk of CVD events or death [13]. Medication adherence is a complex phenomenon that involves patient, clinician and health system factors [14]. This complexity means medication adherence is a difficult outcome to measure effectively as no ‘gold standard’ measurement exists; it has been highlighted that more robust and valid methods for measuring adherence are needed, as well as longer follow-up (12 months or more) evaluation to provide a more realistic understanding of the sustainability of any effect [15]. Moreover, the evidence suggests that different approaches to medication adherence are needed to address the wide heterogeneity in diseases and outcomes [14]. Therefore, the Ready to Reduce Risk (3R) intervention was designed in response to the need for a robust multiple risk factor intervention, primarily aimed at improving medication to statins. It was developed using the established Behaviour Change Wheel [16] framework for designing interventions and the Medical Research Council’s guidance for complex interventions [17] and involves two group education sessions with follow-up text messages and phone support [18].

Following the development of the intervention, we conducted a pragmatic randomised controlled trial in primary care patients already taking statins for the primary prevention of CVD. The primary objective was to evaluate, using an objective biochemical adherence test [12, 19], the impact of the 3R intervention on adherence to statins at 12 months follow-up, when compared to the ‘usual care’ control.

## Methods

### Study design

The study is reported according to the Consolidated Standards of Reporting (CONSORT) checklist [20] (Additional file 1: Fig. S1), and details of the intervention and study have been reported in a published protocol paper [18]. The design was a pragmatic randomised controlled trial with follow-up measures at 12 months. We randomly assigned participants on a 1:1 basis to either the control or intervention group (2 education sessions with follow-up support involving 44 weeks of text messages and 2 phone calls), stratified by age (40–53 years and 54–74 years) and sex. Participants from the same household were automatically allocated to the same group to prevent contamination. Minimal contamination was expected between participants from the same practice [21]. Trained study personnel, not involved in data collection, carried out the randomisation using a secure, computerised software program, following the baseline data collection and confirmation of a total cholesterol level (TC) ≥ 5 mmol/l in line with the eligibility criteria. Blinding of participants was not possible; however, we did take steps to reduce other biases: analysis was undertaken by an independent statistician, and the general practitioners and the urine test team were not informed of a participant’s group allocation. Also, detailed protocol information was not made available until after recruitment to prevent participants from accessing it [22].

The study was designed to be pragmatic, but to mitigate the effects of unbalanced clustering in the intervention group, we used the following: a standardised curriculum and a limited number of facilitators and venues to deliver education, standardised text messages and semi-scripted phone calls. Moreover, we assessed process outcomes relating to fidelity to allow for clear reporting of any variation that occurred, which is in line with the Medical Research Council’s guidance for complex interventions [17].

The design also incorporated a novel, objective biochemical measure of adherence to statins in response to the need for more robust measures. This measure can detect sixty of the most common cardiovascular medications in a spot urine sample and has been shown to be a reliable and practical tool for detecting non-adherence [12, 19] which can be used in conjunction with other measures to assess and improve adherence.

Recruitment and baseline data collection took place between May 2016 and March 2017, with follow-up between May 2017 and April 2018. The study was conducted in collaboration with the Research and Innovation team based at Northampton Healthcare NHS Foundation Trust and was coordinated from the Leicester Diabetes Centre, UK.

### Setting and participants

Participants were recruited from the National Health Service general practices located in the Northamptonshire region of England serving predominately white communities from both rural and urban areas. Practices were approached directly to take part, and lists of potential participants were identified via downloadable automatic searches, based on the eligibility criteria, of the practices’ electronic databases. These were screened by clinical practice staff prior to the mailing of an invitation letter (on practice-headed paper). The invitation letter provided a brief summary about the study and what to do if you wanted to take part. A reply slip with a prepaid envelope was included for the response.

Positive responders were contacted to confirm their interest and their eligibility and to book a slot for the baseline clinic. At this point, an appointment letter with a full patient information sheet was sent out to potential participants to read prior to attendance at the clinic. An appointment reminder text was also sent prior to the clinic to encourage attendance. All positive responders were assigned a study ID, and a log of all screening activity was kept. Eligible participants were asked to attend two nurse-led data collection clinics: at baseline and at 12 months. These were held in a suitable local venue. At the baseline clinic prior to any data collection, the nurse took written informed consent for all participants.

Participants were eligible if they were aged 40 to 74 years old inclusive, had an active statin prescription (at least two issues within the 2 years prior to enrolment) for CVD primary prevention, a total cholesterol (TC) level ≥ 5.0 mmol/l at enrolment, sufficient English language proficiency to participate in the intervention, able to attend study visits, had access to a mobile phone to receive text messages, able to provide informed consent, willing to allow their general practitioner to be notified of the study participation, had no preexisting CVD or inherited lipid disorder and had no established type 1 or type 2 diabetes. Any pregnant women or participants in other clinical intervention studies (within 12 weeks prior to enrolment) were excluded.

### Primary outcome

The primary outcome was medication adherence to statins at 12 months. Participants were asked to provide a urine sample on the days that they attended the data collection clinics. These samples were then sent to a central laboratory where they were analysed by the developers of the biochemical assay measure, which was used to test for the presence of atorvastatin (first choice recommendation for CVD primary prevention [3]), rosuvastatin (a second choice alternative) and common anti-hypertensives. Details of this urine test have been described previously [12, 19].

### Secondary outcomes

Blood samples were taken for non-fasting lipid levels [total cholesterol (TC), high-density lipoprotein cholesterol (HDL-C) and TC-to-HDL ratio] and were sent to and analysed by local laboratories. Other clinical measures were systolic and diastolic blood pressure, weight, body mass index, waist/hip circumference and waist-to-hip ratio. Demographic data (age, sex, ethnicity, smoking status), self-reported medical history (including a family history of CVD) and the number of repeat medications prescribed were recorded. CVD risk scores were calculated from the data collected, using the QRISK2 calculator [6]. Any post-baseline adverse events were reported in accordance with local, standard procedures. Following the 12-month clinic visit, we obtained information from the general practitioners’ databases on the number of prescriptions issued for statins and anti-hypertensives (if applicable) over the 12-month study period, and recorded pre-enrolment cholesterol results and the dates of first statin prescriptions to define our study population. The following self-reported, validated paper questionnaires were used: medication adherence to statins (8-item Morisky Medication Adherence Scale or MMAS which uses a sum of scores equaling either 8, 6 to < 8 or < 6 to categorise adherence as high, medium or low, respectively [23–25]), daily fruit and vegetable intake (Five-A-Day Community Evaluation Tool which records the number of portions consumed [26]), physical activity (short form International Physical Activity Questionnaire which asks 7 questions relating to different physical activities to calculate the time spent, in metabolic equivalent minutes per week, on these activities [27]), patient activation level (short form Patient Activation Measure which uses single-item questions on a 5-point Likert scale which are analysed to give a total score between 0 and 100, with a higher score indicating more activation [28]), health status (Euro Quality of Life 5 Dimensions Questionnaire which asks 5-point Likert scale questions to do with mobility, self-care, usual activities, pain/discomfort and anxiety/depression and records the patient’s self-rated health on a vertical visual analogue scale, with higher scores indicating better health [29]), medication beliefs (Beliefs about Medicines Questionnaire which comprises two 5-point Likert scale question sections: the ‘specific’ which assesses representations of medication prescribed for personal use and the ‘general’ which assesses beliefs about medicines in general, with higher scores indicating increased concerns [30]), health beliefs (Brief Illness Perception Questionnaire uses 10-point questions to assess perceptions about a condition relating to consequence, timeline, personal control, treatment control, identity, concern and emotional representation [31]) and quality of life (15-dimensional questionnaire which comprised questions relating to the quality of activities required for daily living, with a higher overall score on a 0–1 scale indicating a better quality of life [32]).

For any participants not able to attend the 12-month clinic, questionnaires were sent via post and permission was sought to retrieve any relevant clinical data from the general practitioner. A secure, electronic contacts database was used to generate all appointments and to record process outcomes: attendance at clinics and education sessions; initiation, delivery and stopping of text messages; and delivery of follow-up phone calls. In addition, for intervention participants, an anonymous evaluation form was given out following the end of the education sessions to assess the perceived acceptability and usefulness of the sessions.

### Intervention and control groups

The development of the intervention and the theoretical framework has been reported previously [18]. Intervention participants were invited to attend two education sessions and receive follow-up support (44 weeks of text messages and two supportive coaching phone calls). The education involved two group sessions (maximum of eight participants), each lasting about 2 h, in a local community venue. Each session was delivered by two trained facilitators (one of whom was a health professional). Facilitator training involved a 2-day course led by a clinical psychologist plus self-study and a practice run. A written structured curriculum was used to deliver the intervention supplemented by mixed-media educational resources. Session 1 explored understanding and beliefs to do with CVD risk and how to manage it. Participants were shown how to calculate their own risk score by using a CVD risk calculator, prior to exploring factors that influence risk, how these affect the body and the important role that statins have to play in reducing risk. Beliefs about adherence to statins were then explored and how these beliefs are influenced by others such as the media. Session 2 increased knowledge and awareness about how to have a healthier lifestyle to reduce CVD risk and introduced the concept of patient activation [33] and behavioural control techniques (goal setting, action planning, self-monitoring, prompting) to support adherence to statins and a healthy lifestyle.

The support interventions included follow-up text messages and phone calls. An independent text messaging service was set up to deliver a series of automated, unidirectional, personalised text messages providing, primarily, medication reminders to take statins (e.g. “Have you taken your tablets today?”), as well as information, advice and motivation to improve diet, increase physical activity and encourage smoking cessation, if relevant (e.g. “Walking up and down a flight of stairs several times is a great strengthening activity.”). We developed our own medication reminder texts to prompt participants to take their statins each day, and we adapted a bank of messages that had already been robustly developed and validated [34, 35] to deliver the lifestyle information and motivational advice. Texts messages were initiated manually by the study team following the second education session and were delivered on a set schedule, over 44 weeks, that could be stopped at any time by the participant sending a text. Participants were briefed about the text messaging and were also provided with an information booklet. All texts sent were logged and monitored to identify any problems.

For the phone calls, participants were called at home by a trained member of the study team, experienced in dealing with research participants, from a private office using a designated phone. At least three attempts on different days were made to try and contact individuals. Participants received individual calls (at approximately 2 weeks and 6 months) facilitated by a semi-structured script, delivered using a patient-centred approach. Each call lasted approximately 10–20 min, and a written record was kept, using a structured template. Participants were called at a convenient time and questioned about adherence to statins and healthy lifestyle behaviours. Open questions were used to elicit information about what had been going well and not so well, and participants were given the opportunity to discuss any pitfalls and ways to overcome these.

The comparator group and the intervention group continued with their usual general practitioner care for the primary prevention of CVD, which may have involved a CVD risk assessment, a medication review and advice about adhering to statins and healthy lifestyle behaviours. Both groups were given a booklet with general information about CVD risk prevention (‘Keep Your Heart Healthy’ by the British Heart Foundation).

### Sample size

The sample size calculation was based on data about the percentage of non-adherers to cardiovascular medication (including statins) from the Investigation of Text Message Reminders on Adherence to Cardiac Treatment (INTERACT) trial [36]. This study used text messaging, as the sole intervention, and was a 6-month follow-up study with a final control group adherence of 75% and intervention group adherence of 91%, a 16 percentage point difference. At 12 months, we expected similar adherence levels to statin medications following our multiple risk intervention. Therefore, to detect a difference in the proportion of statins adherers (of 16 percentage points in the intervention group, at 12 months, compared with the control group: 91% compared with 75%), we required 84 participants per group with 80% power and 5% significance. After allowing for a 20% dropout, we required 105 participants per group, making 210 participants in total.

### Statistical methods

A statistical analysis plan was finalised and agreed prior to data analysis. We compared participant characteristics by group allocation, using either means (standard deviations) or medians (interquartile ranges) for continuous variables, and counts and percentages for nominal variables. Logistic regression was used to assess the difference in medication adherence by group, adjusted for the stratification factors (sex and age) and baseline adherence at 12 months. The primary outcome was assessed at the 5% level with 95% confidence intervals and primary analysis and was based on complete data with sensitivity analyses carried out on an intention-to-treat and per-protocol basis (defined as participants who had attended at least one group session of the intervention). This was done to examine the robustness of conclusions for missing data and attrition. To adhere to the intention-to-treat principle, missing outcome data were imputed by multiple imputation using the command ‘MI’ in Stata (version 15). This was carried out using logistic regression method with adherence to statin at 12 months as the response variable, adjusted for randomisation, adherence to statins at baseline and stratification factors (sex and age), with 100 imputations. We also conducted a secondary analysis by adding the MMAS adherence data (a high adherence score of 8 defining adherence to statins for this purpose) where urine adherence data was missing. McNemar’s test was used to test the consistency between the two measures where both measures were available. These analyses were also re-run without adjustment for baseline adherence at the request of a reviewer. This analysis was not part of the original analysis plan. The analysis of the secondary outcomes was conducted in a similar manner using the appropriate model type: logistic regression for binary outcomes, linear for continuous outcomes and ordinal for ordinal outcomes. In addition, pre-specified subgroup analyses were performed to look at the effect of the intervention. The number of cardiovascular adverse events was reported.

### Patient and public involvement

Prior to the study, we ran focus groups with patient and public representatives and, in addition, had a patient representative as part of the investigators’ team to provide input on the research question and the study design. This development work highlighted the need for an intervention that not only looked at lifestyle factors but focussed on adherence to statins and led us to have adherence to statins as the primary outcome measure. We sought further feedback from potential patient users throughout the development of the intervention and the study to refine and inform specific elements.

## Results

**Fig. 1:**
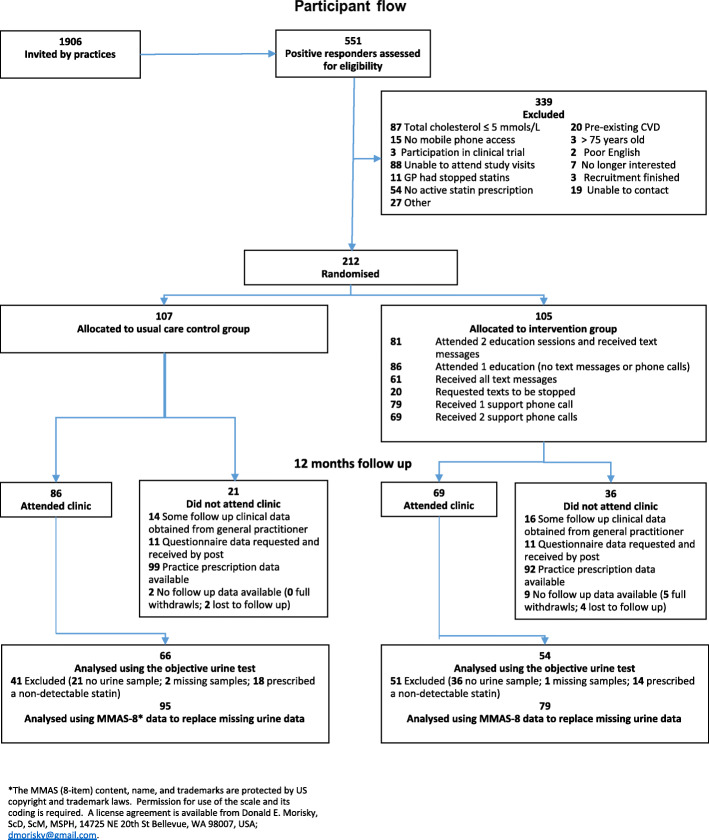
Flow chart

A total of 212 adults were randomised between May 2016 and March 2017. Figure 1 shows the flow of participants through the study. Eleven participants (5%) failed to provide any follow-up data for analysis. Urine data were available for 120 participants (57%), and urine/MMAS-8 data (using MMAS values to replace missing urine values) were available for 174 participants (82%). Eighty-six (82%) of intervention participants attended at least one education session, and 81 (77%) attended both education sessions and received some text messages.

### Baseline characteristics

Table 1 and Additional file 2: Table S1show the participants’ characteristics at baseline. The participants were predominately white ethnicity (*n* = 206 (97%)). There were more women (*n* = 114 (54%)), and the mean age was 63.9 (SD 7.2) years. The mean percentage risk of developing CVD in the next 10 years was 16.2% (SD 8.5), with 60% (SD 28.30) of the participants having a mean percentage CVD risk score greater than 20%. The median number of comorbidities for participants was 2 (interquartile range 1–3). Baseline adherence to statins measured by the urine test was 47% (control *n* = 33) and 62% (intervention *n* = 40). Other participants’ baseline characteristics were generally well balanced across the two study groups. However, the control group was more physically active than the intervention group, although there was no difference between the groups for the total activity. There was only one occurrence of two participants from the same household.

**Table 1:** Baseline characteristics of participants by randomised group: usual care (control) and the 3R intervention. Values are means (standard deviations) unless stated otherwise

Characteristics	Control (*n* = 107)	Intervention (*n* = 105)	All participants (*n* = 212)
Age (years)	63.9 (6.9)	63.9 (7.5)	63.9 (7.2)
Ethnicity (no. (%))			
White	104 (97)	102 (97)	206 (97)
Others	3 (3)	3 (3)	6 (3)
No. (%) women	58 (54)	56 (53)	114 (54)
No. (%) men	49 (46)	49 (47)	98 (46)
Smoking status (no. (%))			
Current	8 (7)	5 (5)	13 (6)
Former	50 (47)	47 (45)	97 (46)
Never	49 (46)	53 (51)	102 (48)
Risk of CVD in next 10 years (mean % (SD))	16 (7.6)	16 (9.4)	16.2 (8.5)
Comorbidities (median (interquartile range))	2 (1–3)	2 (1–3)	2 (1–3)
Biometric measurements			
Body weight (kg)	81.2 (18.4)	81.5 (17.4)	81.3 (17.9)
Body mass index (kg/m^2^)	28.9 (5.1)	28.8 (4.7)	28.9 (5.0)
Waist circumference (cm)	97.3 (13.4)	98.5 (13.0)	97.9 (13.2)
Hip circumference (cm)	106.5 (11.5)	106.2 (9.4)	106.3 (10.5)
Waist-to-hip ratio	0.9 (0.1)	0.9 (0.1)	0.9 (0.1)
Systolic blood pressure (mmHg)	142.0 (18.0)	139.3 (18.1)	140.7 (18.1)
Diastolic blood pressure (mmHg)	87.4 (10.0)	86.1 (10.9)	86.8 (10.4)
Adherences to statins variables			
Urine test for statin adherence (no. (%))	33 (47)	40 (62)	73 (54)
MMAS* score = 8 for high adherence (no. (%))	35 (33)	32 (30)	67 (32)
Total cholesterol (mmol/l)	5.8 (0.8)	5.9 (0.8)	5.9 (0.8)
High density lipoprotein cholesterol (mmol/l)	1.7 (0.6)	1.6 (0.5)	1.7 (0.5)
TC:HDL ratio	3.9 (1.3)	3.9 (1.4)	3.9 (1.3)
No. of years on statins prior to enrolment mean (range)	8.0 (1.2–20.8)	8.3 (1.1–20.6)	8.2 (1.1–20.8)

*TC:HDL* total cholesterol-to-high-density lipoprotein

*Use of the©MMAS is protected by the US and international copyright laws. Permission for use is required. A licence agreement is available from Donald E. Morisky, MMAS Research (MORISKY), 294 Lindura Court, Las Vegas, NV 89138-4632; dmorisky@gmail.com

### Adherence to statins at 12 months (primary outcome)

Table 2 reports adherence to statins by randomisation group and the adjusted odds ratio between the two groups at 12 months follow-up. In the complete case analysis, no differences were found between the control and intervention groups for adherence to statins (urine test) at 12 months (adjusted odds ratio 0.91, 95% confidence interval 0.31 to 2.67). This was confirmed in the sensitivity analyses for both intention-to-treat (adjusted odds ratio 0.79, 95% confidence interval 0.30 to 2.09) and per-protocol analyses (adjusted odds ratio 0.88, 95% confidence interval 0.30 to 2.61). The self-reported MMAS scores and the combination approach of using MMAS data to populate missing urine data values also revealed no differences between the groups for statin adherence (Table 2). McNemar’s test showed a consistency between the urine test measure and the self-report MMAS measure (*P* = 0.006). The value of McNemar’s chi-square was 7.68. When removing the adjustment for baseline adherence, the overall interpretation of the results did not change with no difference between the groups and wide uncertainty.

**Table 2:** Adherence to statins at 12 months follow-up between participants randomised to usual care (control) or the 3R intervention

Variables	Number of participants (%)				Adjusted at follow-up^a^		Adjusted at follow-up*	
	Total**	Control	Total**	Intervention	Odds ratio (95% CI)	*P* value	Odds ratio (95% CI)	*P* value
Complete case^b^								
Statin adherence (urine)	66	36 (55)	54	34 (63)	0.91 (0.31 to 2.67)	0.860	1.37 (0.66 to 2.88)	0.399
Statin adherence (MMAS)	95	36 (38)	79	37 (47)	1.82 (0.89 to 3.74)	0.103	1.46 (0.79 to 2.69)	0.227
Intention to treat^c^								
Statin adherence (urine)	107	62 (57)	105	66 (63)	0.79 (0.30 to 2.09)	0.638	1.24 (0.62 to 2.47)	0.541
Statin adherence (MMAS)	107	39(36)	105	48 (46)	1.81 (0.89 to 3.67)	0.101	1.48 (0.82 to 2.69)	0.193
Per protocol^d^								
Statin adherence (urine)	66	36 (55)	48	31 (61)	0.88 (0.30 to 2.61)	0.824	1.25 (0.59 to 2.64)	0.560
Statin adherence (MMAS)	95	36 (38)	74	36 (49)	1.93 (0.93 to 3.99)	0.076	1.55 (0.83 to (2.90)	0.164
Urine-MMAS data^e^	95	47 (50)	80	46 (58)	0.97 (0.48 to 1.99)	0.945	1.37 (0.75 to 2.50)	0.301

*CI* confidence interval

*Adjusted for stratification factors: sex and age; odds ratio > 1 favours intervention

**Total number includes all participants who had either a urine test or if urine test is not performed, MMAS data available at 12 months

^a^Adjusted for stratification factors: sex and age and baseline value; odds ratio > 1 favours intervention

^b^Participants with missing outcome data or missing variables required for the model adjustment were excluded

^c^Missing data imputed using multiple imputation

^d^Participants who did not engage with at least one group session of the programme have been excluded from the intervention arm

^e^Morisky Medication Adherence Scale (MMAS) was used by adding the MMAS adherence data where urine adherence data were missing, using a high score of 8 to indicate high adherence. Use of the©MMAS is protected by US and international copyright laws. Permission for use is required. A licence agreement is available from Donald E. Morisky, MMAS Research (MORISKY), 294 Lindura Court, Las Vegas, NV 89138-4632; dmorisky@gmail.com

### Secondary outcomes

Table 3 presents the secondary outcomes for the clinical measures at 12-month follow-up, based on a complete case analysis. Differences were adjusted for baseline value and stratification categories (age and sex). Differences between the groups were found for waist circumference (− 2.55 cm) and diastolic blood pressure (− 4.28 mmHg) and systolic blood pressure (− 4.19 mmHg), although the latter was not statistically significant, in favour of the intervention group.

**Table 3:** Changes in clinical measures at 12 months between participants randomised to usual practice (control) or to the 3R intervention

Variable	Number of participants (%)		Mean (SD)		Adjusted difference at follow-up*	
	Control	Intervention	Control	Intervention	Coefficient (95% CI)	*P* value
BMI (kg/m^2^)	86 (80)	69 (66)	28.60 (5.15)	28.29 (4.64)	− 0.36 (− 0.77 to 0.05)	0.088
Body weight (kg)	86 (80)	69 (66)	80.41 (17.43)	79.88 (16.34)	− 0.99 (− 2.12 to 0.13)	0.084
Waist circumference (cm)	86 (80)	69 (66)	99.44 (13.96)	98.22 (13.13)	− 2.55 (− 4.55 to − 0.55)	**0.013**
Hip circumference (cm)	86 (80)	69 (66)	107.39 (10.63)	107.70 (9.75)	0.79 (− 1.14 to 2.73)	0.419
Systolic BP (mmHg)	86 (80)	69 (66)	141.93 (19.04)	137.55 (18.96)	− 4.19 (− 9.13 to 0.76)	0.096
Diastolic BP (mmHg)	86 (80)	69 (66)	85.00 (9.77)	80.52 (10.88)	− 4.28 (− 6.98 to − 1.58)	**0.002**
Total cholesterol (mmol/l)	86 (80)	69 (66)	5.46 (1.01)	5.21 (0.70)	− 0.21 (− 0.46 to 0.05)	0.120
HDL cholesterol (mmol/l)	86 (80)	69 (66)	1.65 (0.59)	1.63 (0.45)	0.01 (− 0.06 to 0.08)	0.814
TC-to-HDL ratio	86 (80)	69 (66)	3.59 (1.14)	3.44 (1.04)	− 0.18 (− 0.40 to 0.05)	0.128
CVD risk score	86 (80)	69 (66)	17.06 (10.30)	16.71 (9.20)	− 1.36 (− 3.28 to 0.56)	0.165
Number of prescriptions	99 (93)	92 (88)	10.80 (3.95)	11.82 (3.62)	0.98 (− 0.07 to 2.04)	0.067

*CI* confidence interval

*Adjusted difference at 12 months between treatment groups with 95% confidence interval, *P* value; adjusted for baseline value and stratification categories (age and sex)

A complete case analysis for adherence to anti-hypertensive revealed no significant differences between the control and intervention groups at 12 months. However, there was a statistically better adherence with a combined total adherence, for participants on both statins and anti-hypertensives, from the urine test measure in favour of the control group (Additional file 3: Table S2).

Table 4 and Additional file 4: Table S3 present the secondary outcomes for the questionnaire measures and the adjusted differences between the groups. A difference between the groups was found for self-reported physical activity (513.65 more minutes per week spent walking for at least 10 min) in favour of the control group. Other differences, in favour of the intervention group scoring more highly on the Brief Illness Perception Questionnaire, were a self-reported likeliness to feel more in control of their treatment and to have a more coherent understanding about their condition.

**Table 4:** Scores for questionnaire measures at 12 months between participants randomised to usual practice (control) or to the 3R intervention

	Number of participants (%)		Mean (SD)		Adjusted difference at follow-up^a^	
	Control	Intervention	Control	Intervention	Coefficient (95% CI)	*P* value
IPAQ^b^ (metabolic equivalent minutes per week)						
Vigorous activity	86 (80)	69 (66)	726.36 (1539.79)	481.90 (1078.52)	− 224.52 (− 574.62 to 125.58)	0.208
Moderate activity	86 (80)	69 (66)	814.02 (1312.92)	903.81 (1529.19)	87.66 (− 300.06 to 475.38)	0.656
Walk at least 10 min	86 (80)	69 (66)	1183.99 (1270.59)	701.96 (874.42)	− 513.65(− 795.19 to − 232.12)	**< 0.001**
Total activity	86 (80)	69 (66)	2724.40 (2623.31)	2087.69 (2350.60)	− 621.72(− 1287.59 to 44.15)	0.067
Brief IPQ^c^						
Consequences	97 (91)	80 (76)	2.71 (2.19)	3.00 (2.40)	0.003 (− 0.54 to 0.55)	0.992
Timeline	97 (91)	80 (76)	7.72 (3.11)	8.48 (2.60)	0.41 (− 0.43 to 1.26)	0.336
Personal control	97 (91)	80 (76)	6.31 (2.55)	6.54 (2.59)	0.31 (− 0.42 to 1.05)	0.402
Treatment control	97 (91)	80 (76)	7.29 (2.20)	7.90 (2.18)	0.66 (0.07 to 1.25)	**0.027**
Identity	97 (91)	80 (76)	3.20 (2.53)	3.32 (2.70)	0.08 (− 0.59 to 0.75)	0.818
Concern	97 (91)	80 (76)	4.21 (2.59)	4.95 (2.95)	0.49 (− 0.25 to 1.23)	0.192
Coherence	97 (91)	80 (76)	7.14 (2.43)	7.82 (2.41)	0.70 (0.07 to 1.33)	**0.030**
Emotional representation	97 (91)	80 (76)	2.69 (2.32)	3.17 (2.78)	0.20 (− 0.37 to 0.77)	0.491
Overall IP score	97 (91)	80 (76)	3.72 (1.39)	3.81 (1.65)	− 0.16 (− 0.51 to 0.19)	0.364

*CI* confidence interval

^a^Adjusted difference for baseline value and stratification categories (age and sex)

^b^International Physical Activity Questionnaire (IPAQ): the amount of metabolic equivalent (MET) minutes = minutes reported per category—× 4 (moderate), × 8 (vigorous) and × 3.3 (walking). Total activity = sum of MET minutes

^c^Brief Illness Perception Questionnaire (IPQ): scale for all responses ranges from 0 to 10. High scores on timeline, consequences, identity, concern and emotional representation reflect a more threatening/poor general health. High scores on the personal control, treatment control and coherence dimensions represent positive beliefs about the controllability of the illness and a personal understanding of the condition

### Subgroup analyses

**Fig. 2:**
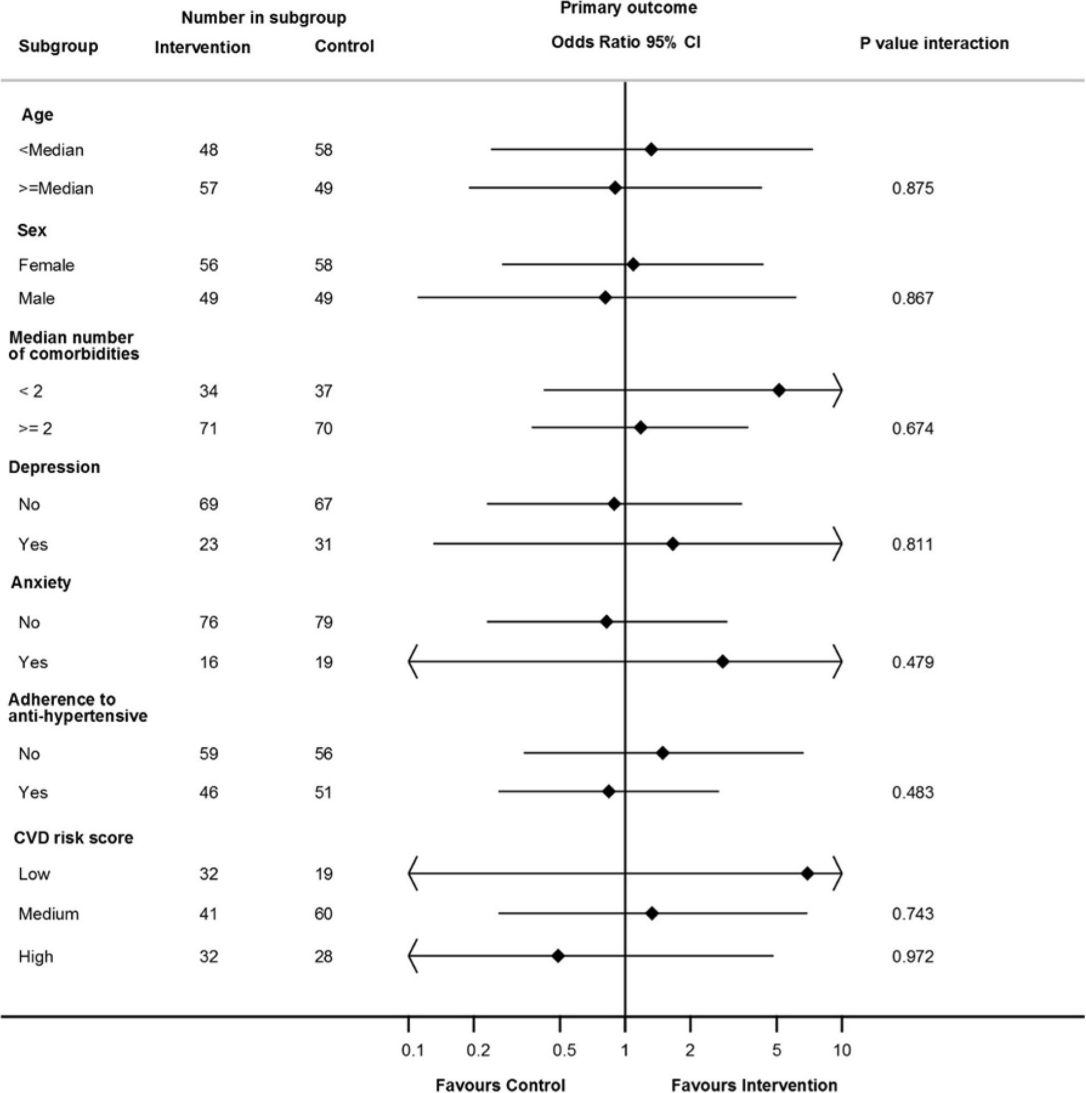
Forest plot of the effect of the 3R intervention at 12 months on the primary endpoints by subgroup

Figure 2 shows the results of the subgroup analyses. No interaction effects were found between the control and intervention groups for the primary outcome.

### Cardiovascular adverse outcomes

A total of four cardiovascular adverse events were reported (2 myocardial infarctions and 1 heart failure in the control group; 1 diagnosis of atrial fibrillation in the intervention group).

### Process outcomes

In the intervention group, 82 feedback forms were completed following the education sessions. The majority of participants felt the education sessions were easy to access (82%), they were given opportunities to speak (73%), the facilitators were friendly and understanding (74%) and support for CVD awareness was given (74%). Regarding behavioural change, 73% felt it was worthwhile and 63% felt it was achievable (Additional file 5: Table S4).

## Discussion

In this pragmatic randomised controlled trial, a multiple risk factor intervention, involving group education with text messaging and telephone support, sought to improve adherence to statins and healthy lifestyle behaviours in people at risk of CVD. At 12 months, there were no differences between the control and intervention groups for adherence to statins, but there were improvements in blood pressure and waist circumference, in favour of the intervention group. Intervention participants felt more in control of and had a more coherent understanding of their condition; additionally, the majority of intervention participants perceived the education sessions as supporting their CVD awareness and felt behavioural change was worthwhile. In comparison, in favour of the control group, there was a difference in physical activity levels for the ‘walk at least 10 min’ per day category and a difference in combined total adherence (adherence to both statins and anti-hypertensives) using the urine test measure.

The study had a number of strengths. It addressed an important research question to find out whether interventions for improving adherence to statins can be successful as part of a multiple risk factor approach. The study also addressed the limitations of previous studies. A novel, objective urine measure was used as the primary measure to detect adherence to statins to offer a more robust and simple way of measuring medication adherence, which did not solely depend on self-report. Furthermore, we measured the effect of the intervention over the longer term of 12 months. The need for longer-term follow-up, to show the sustainability of interventions aimed at improving adherence to statins, has been highlighted previously [15]. Robust methods, theories and guidance were used to design the 3R intervention, and patient involvement was integral to choosing adherence to statins as the focus of the intervention and the primary outcome.

As already noted, the blinding of participants was not possible, but we took steps (where possible) to reduce the risk of bias with regard to the blinding of general practitioners, researchers and participants. We also ensured that the intervention was delivered consistently across the groups. However, as is often the case with pragmatic trials, there were a number of limitations. A large proportion of our study population showed high levels of adherence at baseline, especially in the intervention group, which may have been a key reason as to why the intervention did not lead to an improvement in statin adherence and resulted in a better total combined adherence to both statins and anti-hypertensives in favour of the control group at 12 months. To gain further insight into this issue, a post hoc comparison of baseline characteristics between those participants with and without a urine test was carried out (Additional file 6: Table S5). This revealed that participants with a urine measurement had a greater mean age (+ 2.1 years) and were more likely to have never smoked, showing that selection bias may have occurred in our sample that may explain some of the difference in baseline adherence levels. From an ethical viewpoint, the participants also had to be made aware that their urine was been tested for the presence of statins, which meant that many of the participants might have been more motivated to take their statins prior to the study visit. This ‘toothbrush effect’ is a recognised phenomenon in general practice where patients take their medications prior to a follow-up visit [37]. Thus, the ‘spot check’ urine test might have underestimated non-adherence; although, when compared to the MMAS-8 self-report, the urine biochemical test detected a higher level of non-adherence at baseline (46% versus 40% reporting a low adherence score (< 6) with MMAS-8). The limited accuracy of the MMAS-8 when compared to biochemical medication testing has been demonstrated previously [38]. Therefore, despite its limitations, the urine test would appear to be a more robust and sensitive test for detecting non-adherence than using a self-report measure.

Another limitation of the urine test is that it can only detect the recommended high-intensity statins (e.g. atorvastatin and rosuvastatin). Disappointingly, although we recruited to target, at baseline, 63% of participants were still been prescribed low-intensity statins (e.g. simvastatin 20 mg) which could not be tested for with the urine test—due to the short half-life of simvastatin—and this affected our sample size for analysis. Recent evidence has suggested that patients receiving high-intensity statins, such as atorvastatin, are more likely to be adherent, resulting in larger reductions in cholesterol levels and CVD risk [39]. In our study, as we could only test for the high-intensity statins, this would mean that the urine test was potentially testing the more adherent participants at baseline. There was also a high risk of an attrition bias that was further confounded by the high dropout rates for the 12-month clinics. Our control group was also more physically active at baseline which probably accounted for the improvement in physical activity (walk at least 10 min) result, in favour of the control group. To gain further insight into the reasons for the high dropout rates, we also compared baseline characteristics between those participants who had completed the study with participants who were non-completers (Additional file 7: Table S6). This analysis was not part of the statistical plan. Study completers were more likely to have never smoked and had a lower mean total cholesterol level (− 0.3 mmol/l). This suggests that they may have been the more adherent participants at baseline and is another possible reason as to why the intervention was not effective.

Review evidence suggests that the intensification of patient care (involving strategies such as pharmacist-led interventions, multidisciplinary education or counselling sessions, and automated reminders) can improve both shorter-term and longer-term adherence to statins [15]. Our study showed no significant differences between the groups for adherence to statins following a multiple risk intervention that involved intensifying patient care via group education, text reminders and phone support. However, the intervention group showed improvements in waist circumference and blood pressure when compared to the control group. A previous multiple risk intervention, that involved face-to-face nurse counselling, focused on improving adherence to statins, reducing overweight, smoking cessation and increasing physical activity, with patients’ personal data summarised in a personal risk-factor passport [8]. Following this intervention, the study showed higher adherence levels to statins and lower cholesterol levels for primary prevention patients’ averaged outcomes for 3-, 9- and 18-month time points. A possible reason why our intervention led to improvements in blood pressure and waist circumference, but not in adherence to statins, might be to do with the intervention participants working on maintaining a healthy lifestyle rather than taking their statins, despite the intervention focussing on both these issues. Action planning was used as a behavioural control technique to help participants choose and prioritise specific goals, and it may have resulted in participants focussing more on healthy lifestyle choices rather than medication adherence. In the nurse-led intervention, all participants were initiated on statins, as part of the study, whereas in our study, participants had been prescribed statins for an average of 8 years prior to enrolment. Therefore, there was no real trigger in our study to bring about better statin adherence, and we did not interfere with participants’ statin treatment which, if we had, could have acted as a prompt for better adherence. Intervention participants showed increased levels of control and understanding with regard to their condition, and it appears that they exerted this control in engaging in a more healthy behaviour that led to improved blood pressure and waist circumference. These improvements in blood pressure and waist circumference do fit with review evidence looking at the effect of multiple risk interventions for the primary prevention of CVD [7]. However, the improvement in waist circumference in the intervention group did not see similar improvements in BMI, weight, hip circumference and hip-to-waist ratio, which may suggest a possible measurement error with waist circumference that has skewed this result.

## Conclusion

The 3R multiple risk intervention did not improve adherence to statins for the primary prevention of CVD, but it did lead to improvements in blood pressure and waist circumference, indicating possible engagement in healthy lifestyle behaviours within a pragmatic context which increases the generalisability of the findings and their applicability to usual clinical practice. Our study suggests that, in people who have been on statins for a number of years for the primary prevention of CVD, a broad, multiple risk approach may not be the most effective to bring about improved adherence to statins. In long-term statin users, a better approach to medication adherence would be to target this issue separately so that patients are focussed on changing just this behaviour. Educational interventions, targeting both primary healthcare providers and patients, could be beneficial to ensure that this problem is addressed directly at the point of care, using tools (like the urine test used in this study) to initiate a discussion about the need for statin adherence.

## Supplementary information

**Additional file 1:Fig. S1.** CONSORT checklist.

**Additional file 2:Table S1.** Baseline questionnaire data.

**Additional file 3:Table S2.** Adherence to hypertensives at 12 months.

**Additional file 4:Table S3.** Questionnaires outcomes.

**Additional file 5:Table S4.** Process outcomes.

**Additional file 6:Table S5.** Baseline characteristics of participants between those with and without a urine measurement at 12 months.

**Additional file 7:Table S6.** Baseline characteristics of completers versus non-completers at 12 months.

## Data Availability

The datasets used and/or analysed during the current study are available from the corresponding author on reasonable request.

## Abbreviations

CONSORT: Consolidated Standards of Reporting
CI: Confidence interval
CVD: Cardiovascular disease
HDL-C: High-density lipoprotein cholesterol
LDL-C: Low-density lipoprotein cholesterol
MI: Multiple imputations
MMAS: Morisky Medication Adherence Scale
SD: Standard deviation
TC: Total cholesterol
