# Supplementary material for: Effectiveness of the Ready to Reduce Risk (3R) complex intervention for the primary prevention of cardiovascular disease: a pragmatic randomised controlled trial
Source: BMC Med. 2020 Jul 27;18:198. doi: 10.1186/s12916-020-01664-0 (PMC7384223; doi:10.1186/s12916-020-01664-0)
Supplement: Supplementary file 2 — Additional file 2:Table S1. Baseline questionnaire data. [file 12916_2020_1664_MOESM2_ESM.docx]

| Questionnaires | Control  (n=107) | Intervention  (n=105) | All participants  (n=212) |
| --- | --- | --- | --- |
| Fruit and vegetable intake | 4.8 (2.9) | 4.7 (2.7) | 4.7 (2.8) |
| International Physical Activity Questionnaire (minutes per week) |  |  |  |
| Vigorous activity | 1040.6 (2070.3) | 698.3 (1621.2) | 871.1 (1865.0) |
| Moderate activity | 975.3 (1367.8) | 996.8 (1378.5) | 985.9 (1369.9) |
| Walk at least 10 mins | 1288.1 (1245.9) | 1393.9 (1176.9) | 1340.5 (1210.5) |
| Total | 3304.0 (3194.0) | 3089.0 (2602.5) | 3197.5 (2911.2) |
| Patient Activation Measure | 62.4 (13.1) | 62.9 (13.6) | 62.7 (13.3) |
| Euro Quality of Life 5 Dimensions Questionnaire |  |  |  |
| Index score | 0.8 (0.3) | 0.8 (0.2) | 0.8 (0.2) |
| Visual analogue score | 81.8 (17.2) | 80.0 (16.8) | 81.0 (17.0) |
| Beliefs about Medicines Questionnaire (specific) |  |  |  |
| Concerns | 12.9 (3.9) | 12.5 (4.0) | 12.7 (4.0) |
| Necessity | 15.4 (3.1) | 15.7 (3.2) | 15.6 (3.2) |
| Beliefs about Medicines Questionnaire (general) |  |  |  |
| Overuse | 12.7 (1.9) | 12.5 (2.2) | 12.6 (2.0) |
| Harm | 9.4 (2.3) | 8.8 (2.5) | 9.1 (2.4) |
| Brief Illness Perception Questionnaire: |  |  |  |
| Consequences | 2.7 (2.2) | 3.0 (2.4) | 2.9 (2.3) |
| Timeline | 7.9 (2.8) | 8.6 (2.3) | 8.2 (2.6) |
| Personal control | 6.3 (2.5) | 6.2 (2.6) | 6.2 (2.6) |
| Treatment control | 7.6 (2.1) | 7.6 (2.3) | 7.6 (2.2) |
| Identity | 3.1 (2.5) | 3.0 (2.6) | 3.0 (2.6) |
| Concern | 5.1 (2.7) | 5.3 (2.8) | 5.2 (2.8) |
| Coherence | 6.8 (2.4) | 6.9 (2.7) | 6.8 (2.5) |
| Emotional representation | 2.9 (2.3) | 3.21 (2.76) | 3.04 (2.55) |
| Overall IP score | 3.87 (1.37) | 4.0 (1.5) | 4.0 (1.4) |
| 15-Dimensional Quality of Life Questionnaire | 0.9 (0.1) | 0.1 (0.1) | 0.9 (0.1) |

Supplementary Table 1: Baseline questionnaire results for participants by randomised group: usual practice (control) and 3R intervention. Values are means (standard deviations) unless stated otherwise
