# Supplementary material for: Effectiveness of the Ready to Reduce Risk (3R) complex intervention for the primary prevention of cardiovascular disease: a pragmatic randomised controlled trial
Source: BMC Med. 2020 Jul 27;18:198. doi: 10.1186/s12916-020-01664-0 (PMC7384223; doi:10.1186/s12916-020-01664-0)
Supplement: Supplementary file 3 — Additional file 3:Table S2. Adherence to hypertensives at 12 months. [file 12916_2020_1664_MOESM3_ESM.docx]

**Supplementary Table 2:** Adherence to antihypertensives at 12 months.

|  |  | **Number of particpants (%)** | |  | **Adjusted difference at follow-up ^a^** | |
| --- | --- | --- | --- | --- | --- | --- |
|  |  | **Control** | **Intervention** |  | **Odds Ratio (95% CI)** | **P-value** |
| MMAS ^b^ |  | 95 (55) | 79 (45) |  | 1.82 (0.98 to 3.37) | 0.058 |
| Anti-hypertension adherence ^c^ |  | 29 (27) | 17 (16) |  | 0.68 (0.25 to 1.85) | 0.452 |
| Total adherence ^d^ |  | 65 (61) | 51 (49) |  | 0.34 (0.30 to 3.85) | 0.031 |

^a^ Estimates are adjusted for sex, age, baseline value and the number of medications prescribed; Odds ratio >1 favours intervention

^b^ Morisky Medication Adherence Scale: estimates are derived using proportional odds model for statin adherence. The MMAS (8-item) content, name, and trademarks are protected by US copyright and trademark laws. Permission for use of the scale and its coding is required. A license agreement is available from Donald E. Morisky, ScD, ScM, MSPH, 14725 NE 20th St Bellevue, WA 98007, USA; [dmorisky@gmail.com](mailto:dmorisky@gmail.com).

^c^ Anti-hypertension adherence from urine test

^d^ Total adherence for both statins and anti-hypertensives from urine test.
