# Supplementary material for: Effectiveness of the Ready to Reduce Risk (3R) complex intervention for the primary prevention of cardiovascular disease: a pragmatic randomised controlled trial
Source: BMC Med. 2020 Jul 27;18:198. doi: 10.1186/s12916-020-01664-0 (PMC7384223; doi:10.1186/s12916-020-01664-0)
Supplement: Supplementary file 4 — Additional file 4:Table S3. Questionnaires outcomes. [file 12916_2020_1664_MOESM4_ESM.docx]

|  | Number of participants (%) | | Mean (SD) | | Adjusted difference at follow-up ^a^ | |
| --- | --- | --- | --- | --- | --- | --- |
|  | Control | Intervention | Control | Intervention | Coefficient (95% CI) | P-value |
| Number of daily portions of fruit and vegetable (0 to 4+ ) | 86 (80) | 69 (66) | 4.64 (2.84) | 4.83 (3.06) | 0.17 (-0.72 to 1.07) | 0.702 |
| Patient Activation Measure score^b^ | 97 (91) | 80 (76) | 56.81 (23.79) | 50.31 (31.85) | -6.59 (-14.06 to 0.88) | 0.083 |
| HRQoL 15D ^c^ | 97 (91) | 80 (76) | 0.91 (0.09) | 0.92 (0.09) | 0.01 (-0.001 to 0.02) | 0.074 |
| **EQ-5D^d^** | | | | | | |
| Index score | 97 (91) | 80 (76) | 0.83 (0.23) | 0.85 (0.17) | 0.02 (-0.02 to 0.06) | 0.297 |
| VAS score | 97 (91) | 80 (76) | 81.48 (15.87) | 83.15 (14.61) | 3.24 (-0.37 to 6.85) | 0.078 |
| **BMQ Specific ^e^** | | | | | | |
| Concerns | 97 (91) | 80 (76) | 12.14 (3.91) | 12.04 (3.75) | -0.13 (-1.00 to 0.74) | 0.762 |
| Necessity | 97 (91) | 80 (76) | 15.29 (2.84) | 15.81 (3.25) | 0.30 (-0.36 to 0.96) | 0.367 |
| **BMQ General ^g^** | | | | | | |
| Overuse) | 97 (91) | 80 (76) | 12.47 (1.84) | 12.73 (2.31) | 0.26 (-0.27 to 0.80) | 0.336 |
| Harm | 97 (91) | 80 (76) | 9.11 (2.25) | 8.69 (2.48) | -0.04 (-0.58 to 0.49) | 0.875 |

**Supplementary Table 3:** Scores for questionnaire measures at 12 months between participants randomised to usual practice (control) or to the 3R intervention

^a^ Adjusted difference for baseline value and stratification categories (age and sex).

^b^ Patient Activation Measure: scale for all responses range from 1=strongly disagree to 4=strongly agree and 5=N/A. Higher score indicates higher activation.

^e^ HRQoL (Health Related Quality of Life): a higher 15D index indicating raised health-related quality of life score

^d^ EQ-5D (Euro Quality of Life 5 Dimensions Questionnaire): higher scores indicating good health status.

^f^ BMQ (Beliefs about Medicines Questionnaire) Specific: scale for all responses ranges from 1=strongly disagree to 5=strongly agree with total scores ranging from 5 to 25, and higher scores indicating high concern about potential adverse effects of prescribed medicines, or strong beliefs in necessity and efficacy of prescribed medicines.

^e^BMQ (Beliefs about Medicines Questionnaire) General: scale for all responses ranges from 1=strongly disagree to 5=strongly agree with total scores ranging from 4 to 20, and higher scores indicating strong beliefs that medicines are overused by doctors, or strong beliefs that medicines are harmful.

.
