# Supplementary material for: Effectiveness of the Ready to Reduce Risk (3R) complex intervention for the primary prevention of cardiovascular disease: a pragmatic randomised controlled trial
Source: BMC Med. 2020 Jul 27;18:198. doi: 10.1186/s12916-020-01664-0 (PMC7384223; doi:10.1186/s12916-020-01664-0)
Supplement: Supplementary file 5 — Additional file 5:Table S4. Process outcomes. [file 12916_2020_1664_MOESM5_ESM.docx]

**Supplementary Table 4**: Utility and perceived acceptability of the 3R education programme by intervention participants

| Responses* |  | Feedback forms received =  82 |
| --- | --- | --- |
|  |  | Number of  partipants who agreed/strongly agreed (%) |
| Easy access |  | 67 (82) |
| Well-paced sessions |  | 56 (68) |
| Clear key messages |  | 60 (73) |
| Friendly facilitators |  | 61 (74) |
| Understanding facilitators |  | 61 (74) |
| A relaxed, informal setting |  | 59 (72) |
| Given opportunities to speak |  | 60 (73) |
| Support for CVD awareness given |  | 61 (74) |
| Achievable suggested bahavioural changes |  | 52 (63) |
| Worthwhile suggested behavioural changes |  | 60 (73) |

*Response options were “Strongly agree, agree, uncertain, disagree, strongly disagree” for all 10 items.
