# Supplementary material for: Effectiveness of the Ready to Reduce Risk (3R) complex intervention for the primary prevention of cardiovascular disease: a pragmatic randomised controlled trial
Source: BMC Med. 2020 Jul 27;18:198. doi: 10.1186/s12916-020-01664-0 (PMC7384223; doi:10.1186/s12916-020-01664-0)
Supplement: Supplementary file 6 — Additional file 6:Table S5. Baseline characteristics of participants between those with and without a urine measurement at 12 months. [file 12916_2020_1664_MOESM6_ESM.docx]

**Supplementary Table 5** Baseline characteristics of participants between those with and without a urine measurement at 12 months. Values are means (standard deviations) unless stated otherwise

| Characteristics | With urine measurement  (n=141) | Without urine measurement  (n=71) | All participants  (n=212) | P value |
| --- | --- | --- | --- | --- |
| Age (years) | 64.6 (6.9) | 62.5 (7.6) | 63.9 (7.2) | **0.040** |
| Ethnicity (No (%)): |  |  |  |  |
| White | 137 (97) | 69 (97) | 206 (97) |  |
| Other | 4 (3) | 2 (3) | 6 (3) | 0.993 |
| No (%) women | 74 (52) | 40 (56) | 114 (54) |  |
| No (%) men | 67 (48) | 31 (44) | 98 (46) | 0.595 |
| Smoking status (No (%)): |  |  |  |  |
| Current | 1 (1) | 12 (17) | 13 (6) |  |
| Former | 65 (46) | 32 (45) | 97 (46) |  |
| Never | 75 (53) | 27 (38) | 102 (48) | **<0.001** |
| Biometric measurements: |  |  |  |  |
| Body weight (kg) | 80.7 (16.4) | 82.6 (20.6) | 81.3 (17.9) | 0.479 |
| Body mass Index (kg/m^2^) | 28.7 (4.8) | 29.2 (5.3) | 28.9 (4.9) | 0.535 |
| Waist circumference (cm) | 97.4 (12.9) | 98.8 (13.8) | 97.9 (13.2) | 0.463 |
| Hip circumference (cm) | 106.4 (10.1) | 106.2 (11.3) | 106.3 (10.5) | 0.929 |
| Waist to hip ratio | 0.9 (0.1) | 0.9 (0.1) | 0.9 (0.1) | 0.159 |
| Systolic blood pressure (mm HG) | 139.9 (18.6) | 142.1 (16.9) | 140.7 (18.0) | 0.402 |
| Diastolic blood pressure (mm HG) | 86.5 (9.8) | 87.4 (11.6) | 86.8 (10.4) | 0.530 |
| Total cholesterol (mmol/l) | 5.8 (0.7) | 6.0 (1.1) | 5.9 (0.8) | 0.241 |
| High density lipoprotein cholesterol (mmol/l) | 1.6 (0.5) | 1.7 (0.6) | 1.7 (0.5) | 0.183 |
| TC : HDL ratio | 3.9 (1.3) | 3.8 (1.4) | 3.9 (1.3) | 0.611 |
