# Supplementary material for: Effectiveness of the Ready to Reduce Risk (3R) complex intervention for the primary prevention of cardiovascular disease: a pragmatic randomised controlled trial
Source: BMC Med. 2020 Jul 27;18:198. doi: 10.1186/s12916-020-01664-0 (PMC7384223; doi:10.1186/s12916-020-01664-0)
Supplement: Supplementary file 7 — Additional file 7:Table S6. Baseline characteristics of completers versus non-completers at 12 months. [file 12916_2020_1664_MOESM7_ESM.docx]

**Table S6** Baseline characteristics of completers versus non-completers at 12 months.

Values are means (standard deviations) unless stated otherwise

| Characteristics | Completers  (n=155) | Non-completers  (n=57) | All participants  (n=212) | P value |
| --- | --- | --- | --- | --- |
| Age (years) | 64.4 (7.0) | 62.5 (7.4) | 63.9 (7.2) | 0.086 |
| Ethnicity (No (%)): |  |  |  |  |
| White | 151 (97) | 55 (96) | 206 (97) |  |
| Other | 4 (3) | 2 (4) | 6 (3) | 0.718 |
| No (%) women | 82 (53) | 32 (56) | 114 (54) |  |
| No (%) men | 73 (47) | 25 (44) | 98 (46) | 0.675 |
| Smoking status (No (%)): |  |  |  |  |
| Current | 2 (1) | 11 (19) | 13 (6) |  |
| Former | 71 (46) | 26 (46) | 97 (46) |  |
| Never | 82 (53) | 20 (35) | 102 (48) | **<0.001** |
| Biometric measurements: |  |  |  |  |
| Body weight (kg) | 80.6 (16.2) | 83.4 (21.9) | 81.3 (17.9) | 0.305 |
| Body mass Index (kg/m^2^) | 28.6 (4.7) | 29.5 (5.6) | 28.9 (5.0) | 0.247 |
| Waist circumference (cm) | 97.3 (12.6) | 99.5 (14.6) | 97.9 (13.2) | 0.283 |
| Hip circumference (cm) | 106.1 (9.8) | 107.1 (12.3) | 106.3 (10.5) | 0.529 |
| Waist to hip ratio | 0.9 (0.1) | 0.9 (0.1) | 0.9 (0.1) | 0.316 |
| Systolic blood pressure (mm HG) | 141.2 (19.3) | 139.2 (14.1) | 140.7 (18.0) | 0.474 |
| Diastolic blood pressure (mm HG) | 86.8 (10.6) | 86.8 (9.8) | 86.8 (10.4) | 0.999 |
| Total cholesterol (mmol/l) | 5.8 (0.7) | 6.1 (1.1) | 5.9 (0.8) | **0.033** |
| High density lipoprotein cholesterol (mmol/l) | 1.6 (0.5) | 1.8 (0.6) | 1.7 (0.5) | 0.108 |
| TC : HDL ratio | 3.9 (1.3) | 3.8 (1.3) | 3.9 (1.3) | 0.703 |
